# Feedback control of ErbB2 via ERK-mediated phosphorylation of a conserved threonine in the juxtamembrane domain

**DOI:** 10.1038/srep31502

**Published:** 2016-08-17

**Authors:** Yuki Kawasaki, Ayaka Sakimura, Chul Min Park, Rika Tomaru, Tomohiro Tanaka, Tatsuhiko Ozawa, Yue Zhou, Kaori Narita, Hiroyuki Kishi, Atsushi Muraguchi, Hiroaki Sakurai

**Affiliations:** 1 Department of Cancer Cell Biology, Graduate School of Medicine and Pharmaceutical Sciences, University of Toyama, Sugitani 2630, Toyama 930-0194, Japan; 2 Department of Immunology, Graduate School of Medicine and Pharmaceutical Sciences, University of Toyama, Sugitani 2630, Toyama 930-0194, Japan

## Abstract

Tyrosine kinase activity of the asymmetric EGFR homodimer is negatively regulated via ERK-mediated phosphorylation of Thr-669 in the juxtamembrane domain. In the present study, we investigated in human breast cancer cells whether a similar mechanism plays a role in the feedback regulation of the ErbB2/ErbB3 heterodimer, the most potent ErbB receptor dimer. Constitutive tyrosine phosphorylation of ErbB2 and ErbB3 was significantly decreased in phorbol ester- and growth factor-treated BT-474 and MDA-MB-453 cells. In contrast to the decreased tyrosine phosphorylation, Phos-tag Western blot analysis revealed that TPA induced phosphorylation of ErbB2 in an ERK-dependent manner. The target threonine residue corresponding to EGFR Thr-669 and the surrounding residues are highly conserved in ErbB2, but not in ErbB3. Therefore, we demonstrated ERK-mediated phosphorylation of ErbB2 at Thr-677 by generating phospho-specific monoclonal antibodies. Moreover, treatment with trametinib and SCH772984, inhibitors of the MEK-ERK pathway, and substitution of Thr-677 to alanine impaired the feedback inhibition of ErbB2 and ErbB3. These results demonstrated that ERK-mediated phosphorylation of the conserved threonine is a common mechanism for the negative feedback control of active ErbB receptor dimers.

The ErbB family of receptor tyrosine kinases (RTKs), consisting of epidermal growth factor receptor (EGFR), ErbB2, ErbB3, and ErbB4, triggers intracellular signals, including the mitogen-activated protein kinases (MAPK) and phosphatidylinositol 3-kinase (PI3K)/Akt pathways, and is linked to cell proliferation, differentiation and apoptosis^1^,^2^,^3^,^4^,^5^,^6^,^7^. Overexpression, amplification and mutations of ErbBs are frequently associated with carcinogenesis and malignant progression in many types of human cancers; therefore, anti-ErbB inhibitors have been developed as targeted anti-cancer drugs^6^,^7^,^8^,^9^,^10^,^11^.

In breast cancer cells, ErbB2, an orphan receptor, is frequently overexpressed and forms a heterodimer with a kinase dead counterpart receptor, ErbB3. Heregulin (HRG), a ligand for ErbB3, activates ErbB2/ErbB3, which in turn triggers downstream signals via activation of ErbB2 tyrosine kinase^12^,^13^,^14^. It has been demonstrated that the ErbB2/ErbB3 heterodimer is the most potent receptor dimer combinations among ErbBs in activation of Akt. Lapatinib, a tyrosine kinase inhibitor (TKI) of EGFR and ErbB2, and trastuzumab, an anti-ErbB2 antibody, are used for treatment of breast cancers and some gastric cancers^15^. However, acquired resistance hinders cancer therapy with these agents^16^. Therefore, understanding the regulatory mechanisms of the ErbB family is critical not only for suitable therapy with anti-ErbB inhibitors, but also for overcoming acquired resistance.

We have reported that ligand-independent non-canonical RTK phosphorylation at serine and threonine residues plays crucial roles in receptor signaling, intracellular trafficking, and cancer progression. RSK-mediated Ser-897 phosphorylation of EphA2, for example, regulates cancer cell motility and is related to poor prognosis of lung cancer patients^17^. In addition, EGFR phosphorylated at Thr-669 and Ser-1046/7 via the ERK and p38 pathways, respectively, plays key roles in stress signaling^18^ and *Helicobacter pylori* infection^19^. Recent crystallography studies have revealed that EGFR tyrosine kinase domains form an asymmetric homodimer, consisting of an activator kinase and a receiver kinase, in which the juxtamembrane (JM) domain of the receiver kinase interacts with the C-lobe of the tyrosine kinase (TK) domain of the activator kinase^20^,^21^,^22^,^23^,^24^. In this asymmetric EGFR homodimer, we demonstrated that phosphorylation of the Thr-669 located in the receiver kinase is essential for negative feedback control of constitutive tyrosine phosphorylation^25^.

In the present study, we investigated whether similar feedback control is employed for the ErbB2/ErbB3 heterodimer in breast cancer cells. Because ErbB2 is an orphan receptor and ErbB3 does not have remarkable kinase activity, it is thought that ErbB2 and ErbB3 function as the receiver and activator, respectively, in their heterodimeric forms. We investigated whether similar mechanisms are involved in the feedback inhibition of the ErbB2/ErbB3 heterodimer via ERK-mediated phosphorylation of the conserved threonine in the ErbB2 receiver kinase.

## Results

### Downregulation of constitutive ErbB2 and ErbB3 tyrosine phosphorylation

**Figure 1:**
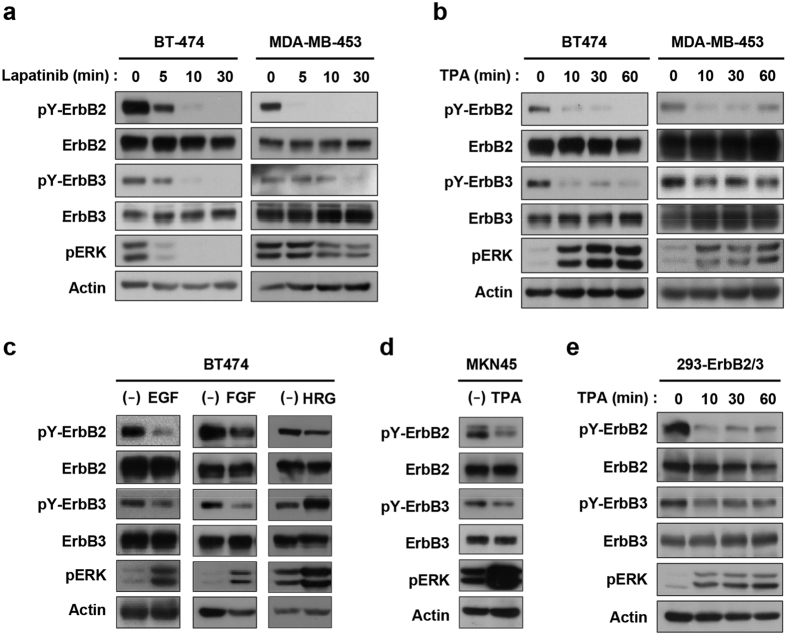
Downregulation of ErbB2 and ErbB3 tyrosine phosphorylation. BT-474 and MDA-MB-453 cells were treated with 1 μM lapatinib (**a**) or 100 ng/ml TPA (**b**) for the indicated time. (**c**) BT-474 cells were stimulated with 10 ng/ml EGF, FGF2, or 50 ng/ml HRG for 10 min. (**d**,**e**) MKN-45 cells and HEK293 cells stably expressing both ErbB2 and ErbB3 (293-ErbB2/3) were treated with TPA for 10 min. Whole cell lysates were subjected to immunoblotting with antibodies against phospho-specific ErbB2 Tyr-1196 (pY-ErbB2), ErbB3 Tyr-1289 (pY-ErbB3), ErbB2, ErbB3, phospho-ERK (pERK), and Actin. The difference of pERK band intensity in each non-treated cells was due to the different exposure time between experiments.

We previously reported that ERK activation in MDA-MB-468 cells results in the downregulation of tyrosine autophosphorylation of the EGFR homodimer^25^. Here, we investigated the possibility of whether such feedback mechanisms are utilized for the ErbB2/ErbB3 heterodimer. We first confirmed ErbB2/ErbB3 hererodimerization in ErbB2-overexpressing BT-474 and MDA-MB-453 human breast cancer cells. Lapatinib, an ErbB2 tyrosine kinase inhibitor, efficiently inhibited constitutive phosphorylation of ErbB3 at Tyr-1289 (pY-ErbB3) as well as ErbB2 at Tyr-1196 (pY-ErbB2), major autophosphorylation sites, indicating their functional heterodimerization (Fig. 1a). Similar to a previous report using trastuzumab (ref. 26), pERK was completely inhibited by lapatinib in BT474 cells, indicating that downstream signaling is highly dependent on ErbB2 activity in BT474 cells (Fig. 1a). Treatment with TPA resulted in ERK activation and subsequent decreases in the constitutive tyrosine phosphorylation of both pY-ErbB2 and pY-ErbB3 (Fig. 1b). Similarly, tyrosine phosphorylation of these ErbBs was also decreased by EGF and FGF2 (Fig. 1c). HRG, an ErbB3 ligand, also downregulated pY-ErbB2, and increased pY-ErbB3 (Fig. 1c). ErbB2 inhibitors have recently been approved for the treatment of ErbB2-positive gastric cancers; therefore, we next investigated the feedback control of ErbB2 in MKN45 human gastric cancer cells. Figure 1d shows similar TPA-induced inhibition of constitutive pY-ErbB2 and pY-ErbB3. To characterize the ErbB2/ErbB3 heterodimer, we established HEK293 cells stably expressing both ErbB2 and ErbB3 (293-ErbB2/3), and observed TPA-induced downregulation of pY-ErbB2 and pY-ErbB3 without change in total protein expression (Fig. 1e). However, subcellular localization of ErbB2 was not significantly changed in TPA-treated BT-474 cells (Supplemental Fig. S1). Together, these results clearly demonstrated that activation of the ErbB2/ErbB3 heterodimer is regulated in a similar fashion to the feedback control of the EGFR homodimer.

### Involvement of phosphatases in tyrosine dephosphorylation

**Figure 2:**
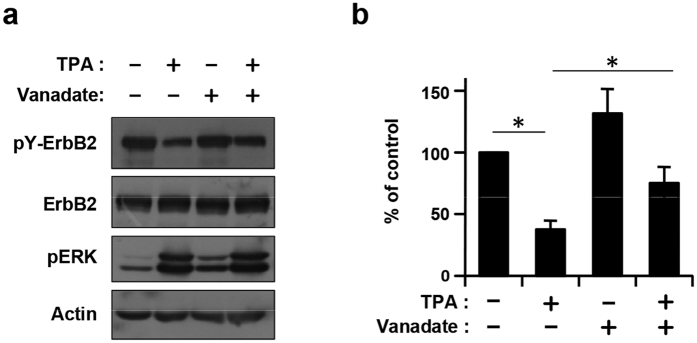
Effect of vanadate on ErbB2 tyrosine phosphorylation. (**a**) 293-ErbB2/3 cells were pretreated with 1 mM vanadate for 15 min, and then treated with TPA for 10 min. Whole cell lysates were subjected to immunoblotting with antibodies against pY-ErbB2, ErbB2, pERK, and Actin. (**b**) Band densities were determined and the level of pY-ErbB2 was calculated as a ratio against total ErbB2 quantity. Values (% of control) represent mean ± S.D. from four independent experiments, *p < 0.01.

The downregulation of pY-ErbB2 was observed within ten minutes of lapatinib treatment or TPA stimulation (Fig. 1a,b). These results suggested that constitutive dephosphorylating activity is present in cancer cells. We therefore tested the effects of vanadate, a non-selective tyrosine phosphatase inhibitor, on tyrosine dephosphorylation feedback control. Figure 2 shows that downregulation of pY-ErbB2 was significantly restored in the presence of the inhibitor, indicating the involvement of vanadate-sensitive tyrosine phosphatases in the negative feedback inhibition of ErbB2.

### ERK-mediated phosphorylation of ErbB2 and ErbB3

**Figure 3:**
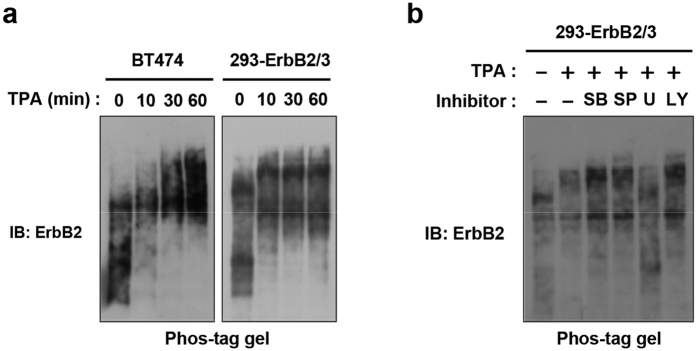
ERK-mediated phosphorylation of ErbB2. (**a**) BT-474 and 293-ErbB2/3 cells were treated with 100 ng/ml TPA, and whole cell lysates were subjected to Phos-tag Western blotting with an anti-ErbB2 antibody. (**b**) 293-ErbB2/3 cells were pretreated with 10 μM SB203580, 10 μM SP600125, 10 μM U0126, or 10 μM LY294002 for 30 min, and then treated with 100 ng/ml TPA for 10 min. Whole cell lysates were subjected to Phos-tag Western blotting with an anti-ErbB2 antibody.

Alternations of ErbB2/ErbB3 phosphorylation status were examined by Phos-tag Western blotting, a recently developed assay system that detects phosphorylated proteins as shifted bands. TPA clearly induced several shifted bands of ErbB2 in BT-474 and 293-ErbB2/3 cells (Fig. 3a), indicating that the total phosphorylation status of ErbB2 was increased, despite the reduction in tyrosine phosphorylation level (Fig. 1b,e). In addition, U0126, an MEK inhibitor, but not JNK, p38 and PI3K inhibitors, restored these band shifts (Fig. 3b). These results suggested that the ERK pathway induces ErbB2 phosphorylation at multiple sites, possibly including Ser/Thr residues, in the TPA-induced feedback inhibition.

### Phosphorylation of ErbB2 Thr-677 regulates the downregulation of ErbB2 and ErbB3

**Figure 4:**
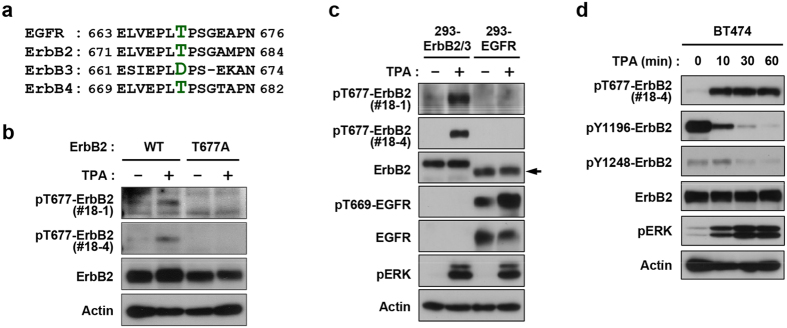
TPA-induced phosphorylation of ErbB2 at Thr-677. (**a**) Alignment of amino acid sequences around EGFR Thr-669 among four ErbB members is shown. Amino acids corresponding to EGFR Thr-669, ErbB2 Thr-677 and ErbB3 Asp-667 are shown in green. (**b**) HEK293 cells were transiently transfected with expression plasmids for wild type (WT) and Thr-677-Ala mutant (T677A) ErbB2. Twenty-four hours after transfection, cells were treated with TPA for 10 min. (**c**) 293-ErbB2/3 and 293-EGFR cells were treated with TPA for 10 min. The arrowhead shows cross reactivity to EGFR. (**d**) BT-474 cells were treated with TPA for the indicated time. Whole cell lysates were subjected to immunoblotting with antibodies against phospho-ErbB2 (Thr-677) (clones No. 18-1 and 18-4), phospho-ErbB2 (Tyr-1196 and Tyr-1248), ErbB2, phospho-EGFR (Thr-669), EGFR, phospho-ERK, and Actin.

The amino acid sequence of the feedback site of EGFR around Thr-669 is highly conserved in ErbB2, including Thr-677 (Fig. 4a). In contrast, the threonine phosphorylation residue is replaced by aspartic acid in ErbB3 (Fig. 4a). These observations suggest that ErbB2 is the main target kinase in the feedback control of the ErbB2/ErbB3 heterodimer. Therefore, we generated phospho-ErbB2 (Thr-677) antibodies using the immunospot array assay on a chip (ISAAC) technology, a rapid isolation system for rabbit monoclonal antibodies^27^,^28^. We obtained two phospho-specific clones available for Western blot, 18-1 and 18-4. The K_d_ values of 18-1 and 18-4 were 2.17 × 10^−9^ M and 2.54 × 10^−9^ M, respectively (Supplemental Fig. S2). Both antibodies reacted with wild type ErbB2, but not with the Thr-677-to-Ala substitution mutant, in TPA-treated 293 cells (Fig. 4b). Most importantly, these antibodies selectively detected phosphorylated ErbB2, but not Thr-669-phosphorylated EGFR (Fig. 4c). In a time course analysis, phosphorylation of Thr-677 (pT-ErbB2) in TPA-treated BT-474 cells was rapidly induced within 10 min in parallel to ERK activation and these changes were inversely correlated with phosphorylation of ErbB2 at two different tyrosine residues, including Tyr-1196 and Tyr-1248 (Fig. 4d).

### Thr-677 is a feedback phosphorylation site of ErbB2

**Figure 5:**
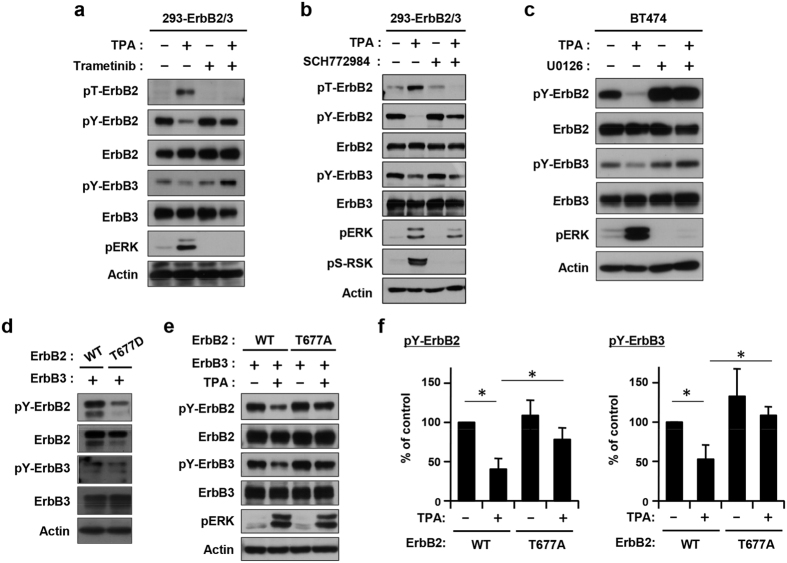
Feedback inhibition of ErbB2/ErbB3 via ERK-mediated phosphorylation of ErbB2. (**a**) 293-ErbB2/3 cells were pretreated with trametinib (0.03 μM), and then treated with TPA for 10 min. (**b**) 293-ErbB2/3 cells were pretreated with SCH772984 (0.5 μM), and then treated with TPA for 10 min. (**c**) BT-474 cells were pretreated with U0126 (10 μM), and then treated with TPA for 10 min. (**d**) HEK293 cells were transiently transfected with ErbB2 (WT or T677D) and ErbB3. (**e**) HEK293 cells transiently transfected with ErbB2 (WT or T677A) and ErbB3 were treated with TPA for 10 min. Whole cell lysates were subjected to immunoblotting with antibodies against phospho-ErbB2 (Thr-677) (clone No. 18-4), pY-ErbB2 (Tyr-1196), ErbB2, pY-ErbB3, ErbB3, p-ERK, and Actin. (**f**) Band densities were determined and the level of pY-ErbB2 and pY-ErbB3 were calculated as a ratio against total ErbB2 and ErbB3 quantity, respectively. Values (% of control) represent mean ± S.D. from five independent experiments, *p < 0.05.

We assessed the role of ERK-mediated phosphorylation of Thr-677 in the feedback inhibition of the ErbB2/ErbB3 heterodimer. Trametinib, a recently approved MEK inhibitor, completely blocked TPA-induced ERK activation and subsequently pT-ErbB2, confirming that phosphorylation of Thr-677 was regulated by the MEK-ERK pathway. This is consistent with the band shifts observed in the Phos-tag gel shown in Fig. 2. In addition, downregulation of the ERK-ErbB2 pathway by trametinib abrogated TPA-induced downregulation of both pY-ErbB2 and pY-ErbB3 (Fig. 5a). Furthermore SCH772984, a newly developed ERK inhibitor (ref. 29), abolished both TPA-induced phosphorylation of Thr-677 and downregulation of pY-ErbB2 and pY-ErbB3 (Fig. 5b). U0126, another MEK inhibitor, also abolished the feedback inhibition of ErbB2 and ErbB3 in BT-474 cells (Fig. 5c). To obtain direct evidence for the role of the conserved threonine, amino acid-substituted ErbB2, phospho-mimic Thr-677-Asp (T677D) and phospho-deficient Thr-677-Ala (T677A) mutants, were transiently expressed with wild type ErbB3 in HEK293 cells. ErbB2-T677D reduced the level of pY-ErbB2 and pY-ErbB3 (Fig. 5d) without TPA stimulation. By contrast, ErbB2-T677A was largely resistant to TPA-induced feedback inhibition of both pY-ErbB2 and pY-ErbB3, although ERK was activated (Fig. 5e). Quantitative analysis demonstrated significant differences in the downregulation of pY-ErbB2 and pY-ErbB3 (Fig. 5f).

### Effect of feedback control of ErbB2 on Akt activation

**Figure 6:**
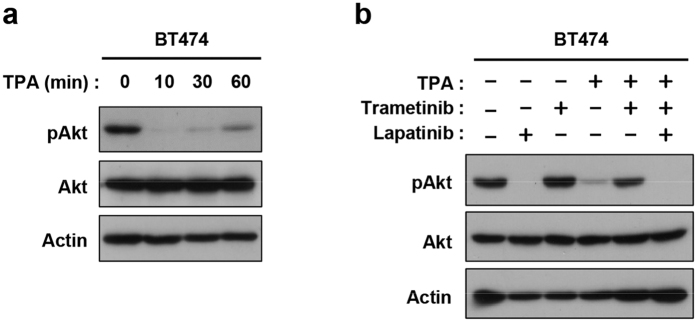
Downregulation of Akt by TPA-induced ERK activation. (**a**) BT-474 cells were treated with TPA for the indicated time. (**b**) BT-474 cells were pretreated with lapatinib (1 μM) and/or trametinib (0.03 μM), and then treated with TPA for 10 min. Whole cell lysates were subjected to immunoblotting with antibodies against phospho-Akt (Ser-473), Akt, and Actin.

**Figure 7:**
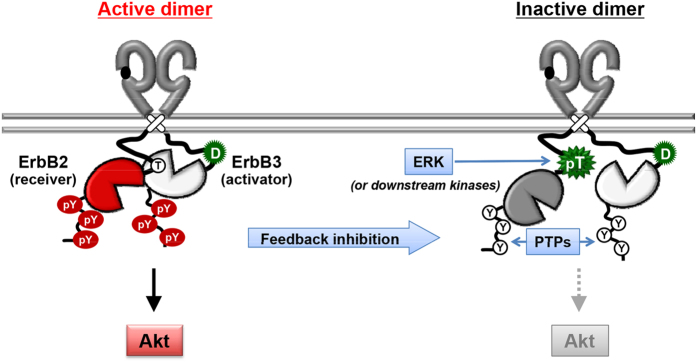
Schematic diagram of the feedback control of the ErbB2/ErbB3 heterodimer. Formation of a dimer structure by interaction of the N-lobe of the receiver kinase (ErbB2) with the C-lobe of the activator kinase (ErbB3) induces phosphorylation of tyrosine residues (pY) in the C-tails of both ErbB2 and ErbB3. Aspartic acid (D) in ErbB3 may restrict ErbB3 interaction with the receiver side. ERK-mediated Thr-677 phosphorylation of ErbB2 (pT) induces conformational change to disrupt the active dimer structure, which leads to tyrosine dephosphorylation in the C-terminal by protein tyrosine phosphatases (PTPs) and suppression of Akt activation.

Finally, we examined the effect of ErbB2 downregulation on Akt activation in BT-474 cells. Constitutive Akt phosphorylation was inhibited by lapatinib, indicating ErbB2-dependent activation (Fig. 6b). Treatment with TPA also resulted in rapid inhibition of Akt activation (Fig. 6a). In addition, TPA-induced suppression of Akt was restored by pretreatment with trametinib (Fig. 6b). These results suggested that ERK-mediated phosphorylation of ErbB2 Thr-677 is critical for feedback inhibition of the ErbB2/ErbB3 heterodimer and their downstream Akt pathway (Fig. 7).

## Discussion

The activation mechanisms of the EGFR/ErbB receptors have been extensively studied; however, it is also important to understand the molecular mechanisms for their inactivation. Receptor endocytosis and subsequent lysosomal degradation is one major pathway for termination of ErbB activation^30^,^31^. In addition, dephosphorylation of tyrosine residues is another mechanism underlying the inactivation of ErbB receptors^12^; however, the details of this mechanism remain to be clarified.

Furthermore, it remains unknown whether dephosphorylation is passively catalyzed by protein tyrosine phosphatases or if there are some mechanisms that actively trigger dephosphorylation. Figure 1a shows that treatment of lapatinib resulted in dephosphorylation of ErbB2 and ErbB3 within 10 min, without protein degradation. Rapid dephosphorylation of EGFR was also observed in gefitinib-treated lung cancer cells^25^. These results indicate that strong dephosphorylating activity is present constitutively in cancer cells and inhibition of TK activity is sufficient to rapidly increase the number of unphosphorylated receptors. On the other hand, we have demonstrated that ERK-mediated phosphorylation of the conserved threonine residues in the JM domain induces tyrosine dephosphorylation of EGFR and ErbB2. This feedback control of ErbB receptors via downstream kinases suggested the existence of actively controlled mechanisms for its downregulation. However, it remains unknown whether threonine phosphorylation accelerates the access of protein tyrosine phosphatases (PTPs) to the phosphorylated tyrosines, or directly inhibits the TK activity of ErbB receptors. Involvement of PTPs, such as PTPN12, PTPN13, and PTPN18, in tyrosine dephosphorylation of ErbB2 has been reported^32^,^33^,^34^. Several other downregulators of ErbBs, including Mig-6, have been characterized, suggesting the presence of active regulatory mechanisms for the termination of TK activity and maintenance of the inactive state via activation of the ERK pathway^35^.

Structural insights into this feedback control may clarify the role of target threonine phosphorylation in the JM domain (Fig. 7). Structure analyses of the asymmetric EGFR homodimer have shown that the JM domain of the receiver kinase plays a key role in maintaining the active conformation by interacting with the C-lobe of the activator kinase. In particular, a small segment, from Leu-664 to Ser-671, of the receiver kinase (called the JM latch) is important for interaction with the activator kinase^23^,^24^. In addition, kinase activity is not essential for the activator function; therefore, kinase-dead ErbB3 is known to participate only as an activator in the ErbB dimers. In the present study, we demonstrated that, similar to EGFR Thr-669, Thr-677 in the JM domain of ErbB2 is a target site of ERK for the feedback inhibition of the ErbB2/ErbB3 heterodimer. It is interesting that the corresponding residue in ErbB3 is replaced with aspartic acid (Asp-667), which has an acidic charge that mimics phosphorylated threonine. This evidence suggested that the JM domain containing Asp-667 may structurally prevent free interaction of ErbB3 with the receiver side of the ErbB heterodimer. Similar to EGF and FGF, ErbB3 ligand HRG induced negative feedback regulation of ErbB2, although tyrosine autophosphorylation of ErbB3 was significantly increased (Fig. 1c). We previously demonstrated that Thr-669 in the receiver kinase, but not the activator, controls feedback inhibition of the EGFR homodimer^25^. These results support the idea that receiver kinase ErbB2, but not activator ErbB3, plays a role in the feedback regulation of the ErbB2/ErbB3 heterodimer. Altogether, these observations revealed that structural changes in the JM latch by introducing an acidic charge may disrupt the active asymmetric conformation of the two kinase domains, which allow PTPs to rapidly dephosphorylate the tyrosine residues.

We used Phos-tag gel for detecting total phosphorylation levels of ErbB2 (Fig. 3). Some shifted and smear bands were observed upon stimulation with TPA and large parts of the band shift were restored by U0126, indicating that ErbB2 is mainly phosphorylated by the MEK-ERK pathway. In the present study, we focused on Thr-677 in the JM domain as a main negative feedback site; however, there is a possibility that other Ser/Thr sites are collaborated with Thr-677. In fact, T677A mutation of ErbB2 did not completely suppressed TPA-induced dephosphorylation of ErbB2 and ErbB3 (Fig. 5d,e). Therefore, characterization of other phosphorylation sites and their physiological functions is essential for full understanding of the activation and inactivation mechanisms for ErbB2 in cancer cells.

From a clinical point of view, bypassing RTK signaling pathways is one of the major causes of acquired resistance to anticancer kinase inhibitors, including MEK inhibitors^7^,^36^. The ERK and Akt pathways have been characterized as two major signaling pathways that support cancer cell proliferation and survival. Trametinib, a MEK inhibitor, strongly suppresses activation of ERK, which leads to compensatory activation of Akt via activation of oncogenic RTKs^37^. In addition, Chung *et al.* also reported that TPA induced downregulation of pAkt in lung adenocarcinoma cells^38^. All together, it is possible that suppression of ERK results in the release of EGFR and ErbB2 from negative feedback inhibition by dephosphorylation of the JM domain. Therefore, further characterization of the feedback mechanisms of RTKs may provide information on how to overcome such resistance. Immunohistochemical analysis of the phospho-threonine levels of EGFR and ErbB2 is also beneficial for understanding the roles of non-canonical feedback phosphorylation in resistant tumors.

In summary, we found that the conserved threonine residue in the JM region contributes to negative feedback inhibition of the constitutive tyrosine phosphorylation of ErbB receptor dimers. In phosphorylation site databases, multiple uncharacterized serine and threonine residues are deposited as possible phosphorylation sites; therefore, it is essential to further investigate the functional significance of non-canonical serine/threonine phosphorylation of the ErbB receptors.

## Methods

### Materials

Phospho-specific antibodies against ERK (Thr-202 and Tyr-204), Akt (Ser-473), p90RSK (Ser-380), EGFR (Thr-669), ErbB2 (Tyr-1196 and Tyr-1248) and ErbB3 (Tyr-1289), and total EGFR antibody were purchased from Cell Signaling Technology (Danvers, MA, USA). Antibodies against EGFR, ErbB2, ErbB3, Akt1 and Actin were purchased from Santa Cruz Biotechnology (Santa Cruz, CA, USA). Recombinant human epidermal growth factor (EGF), heregulin (HRG), and fibroblast growth factor-2 (FGF-2) were purchased from R&D Systems (Minneapolis, MN, USA). SB203580, SP600125, U0126, and LY294002 were obtained from Merck Biosciences (Darmstadt, Germany). Lapatinib and trametinib were obtained from AdooQ BioScience (Irvine, CA, USA) and Cayman Chemical (Ann Arbor, MI, USA), respectively. SCH772984 was purchased from Chemietek (Indianapolis, IN, USA). TPA and Phos-tag ligand were purchased from WAKO Pure Chemical Industries (Osaka, Japan). All chemical agents were dissolved in DMSO, and the final concentration of DMSO was less than 0.1%.

### Cell culture

BT-474, MBA-MD-453, and HEK293 cells were maintained in Dulbecco’s modified Eagle medium containing 10% FCS, 2 mM glutamine, 100 U/ml penicillin, and 100 μg/ml streptomycin at 37 °C with 5% CO_2_ in air. MKN45 cells were maintained in RPMI1640 medium containing 10% FCS, 2 mM glutamine, 100 U/ml penicillin, and 100 μg/ml streptomycin at 37 °C with 5% CO_2_ in air. HEK293 cells stably expressing EGFR (293-EGFR)^25^ and ErbB2/ErbB3 (293-ErbB2/ErbB3) (see below) were maintained in the presence of G418.

### Transfection and establishment of HEK293-ErbB2/ErbB3 cells

Human ErbB2 and ErbB3 cDNAs were inserted into the expression vector pcDNA3.1 (Life Technologies, Carlsbad, CA, USA). KOD FX neo polymerase (Toyobo, Osaka, Japan) and PrimeSTAR polymerase (TaKaRa Bio, Shiga, Japan) was used for substitution of Thr-677 to Ala and Asp, respectively, in ErbB2. Plasmid DNAs were transfected into HEK293 cells using Lipofectamine 2000 (Life Technologies). Stable cell lines were established in media containing 1 mg/ml G418.

### Western blotting

Cells were treated with the reagents in serum-containing media as described above and then lysed in whole cell lysis buffer for normal blotting (25 mM HEPES-NaOH (pH 7.7), 0.3 M NaCl, 1.5 mM MgCl_2_, 0.2 mM EDTA, and 0.1% Triton X-100) or RIPA buffer for Phos-tag blotting (50 mM Tris-HCl (pH 7.4), 0.15 M NaCl, 0.25% sodium deoxycholate, 1.0% NP-40, 1 mM EDTA) containing 1 mM dithiothreitol, 10 μg/ml aprotinin, 10 μg/ml leupeptin, 1 mM sodium orthovanadate, 20 mM β-glycerophosphate, and 1 mM phenylmethylsulfonyl fluoride. SDS-PAGE or Zn^2+^-Phos-tag SDS-PAGE were performed, as described previously^25^,^39^, and the proteins were transferred onto an Immobilon-P nylon membrane (Millipore, Billerica, MA, USA). The membrane was blocked with BlockAce (Dainippon Sumitomo Pharmaceutical, Osaka, Japan), incubated with primary antibodies described above, and then incubated with a horseradish peroxidase-conjugated anti-rabbit, anti-mouse, or anti-goat IgG secondary antibody (Dako, Glostrup, Denmark). Signals were visualized using the ECL system (Life Technologies).

### Generation of rabbit monoclonal antibodies against pT677-ErbB2

Monoclonal antibodies against phospho-ErbB2 Thr-677 were generated using the rabbit-immunospot array assay on a chip (ISAAC) system, as described previously^27^,^28^. The synthetic peptides, ErbB2-peptide (EPLTPSGAMP), ErbB2-peptide phosphorylated at Thr-677 (pT-ErbB2; EPL(pT)PSGAMP), biotinylated pT-ErbB2 peptide, and KLH conjugated pT-ErbB2 peptide, were obtained from Eurofins (Tokyo, Japan). A rabbit was immunized with the KLH-conjugated pT-ErbB2 peptide. Immunoglobulin (IgG) was purified, titrated by an enzyme-linked immunosorbent assay, and applied for Western blotting. The antibody affinities (K_d_) were determined as described previously^27^. Experiments using rabbits were approved by the Committee on Animal Experiments at University of Toyama and were carried out in accordance with the approved guidelines.

### Data processing and statistical analysis

We confirmed the reproducibility of the data in more than two independent experiments and a representative result is shown. Quantitative analysis was made by densitometry using ImageJ software. Statistically differences were determined by one-way analysis of variance and Tukey-Kramer HSD test. *P* values of <0.05 were considered as significant.

## Additional Information

**How to cite this article**: Kawasaki, Y. *et al.* Feedback control of ErbB2 via ERK-mediated phosphorylation of a conserved threonine in the juxtamembrane domain. *Sci. Rep.* **6**, 31502; doi: 10.1038/srep31502 (2016).

## Supplementary Material

Supplementary Information
