# Supplementary material for: Feedback control of ErbB2 via ERK-mediated phosphorylation of a conserved threonine in the juxtamembrane domain
Source: Sci Rep. 2016 Aug 17;6:31502. doi: 10.1038/srep31502 (PMC4987620; doi:10.1038/srep31502)
Supplement: Supplementary Information [file srep31502-s1.doc]

**Supplementary Information**

**Feedback control of ErbB2 via ERK-mediated phosphorylation of a conserved threonine in the juxtamembrane domain**

Yuki Kawasaki, Ayaka Sakimura, Chul Min Park, Rika Tomaru, Tomohiro Tanaka, Tatsuhiko Ozawa, Yue Zhou, Kaori Narita, Hiroyuki Kishi, Atsushi Muraguchi, Hiroaki Sakurai

**Supplementary Figure S1. Subcellular distribution of ErbB2.** BT-474 cells were stimulated with TPA for the indicated time, and immunofluorescence analysis was carried out using anti-ErbB2 antibody, Alexa Fluor 488-conjugated goat anti-rabbit IgG antibody (Life Technologies) and DAPI. Fluorescence was analyzed by LSM700 confocal microscopy (Zeiss).

**Supplementary Figure S2. Characterization of pT-ErbB2 rabbit monoclonal antibodies (RaMoAbs).** (A) Specificity of RaMoAbs was examined by ELISA using pT-ErbB2 peptide- or ErbB2 peptide-coated 96-well plate. (B) 293-ErbB2/3 cells were treated with TPA for 10 min, and whole cell lysates were immunoblotted with RaMoAbs in the absence or presence of competitor peptides (10 μg/ml). pT-ErbB2 band was not competed by non-phosphorylated peptide (T). In contrast, Thr-677-phosphorylated antigen peptide (pT) completely blocked the binding to ErbB2. (C) The antibody affinity for pT-ErbB2 was determined. pT-ErbB2 RaMoAbs (0.1 nM) were incubated with pT677-ErbB2 peptide (0.6 to 150 nM). The concentration of free antibody that remains unsaturated at equilibrium was measured by ELISA using pT-ErbB2-peptide-coated 96-well plate. Data are a representative of three independent experiments with similar results. The data were used for determination of Kd values using Scatchard plots.
